# Mindfulness in Adaptation to Bereavement: A Systematic Review

**DOI:** 10.1002/cpp.70198

**Published:** 2025-12-11

**Authors:** Xinyan Sun, Maya J. Schroevers, Max O. Lessing, Julie Karsten, Maarten C. Eisma

**Affiliations:** ^1^ Department of Health Sciences, Health Psychology Section University of Groningen/University Medical Center Groningen Groningen the Netherlands; ^2^ Department of Clinical Psychology and Experimental Psychopathology University of Groningen Groningen the Netherlands

**Keywords:** anxiety, depression, mindfulness‐based interventions, posttraumatic stress disorder, prolonged grief disorder, self‐reported mindfulness

## Abstract

Bereavement can precipitate mental health problems, including severe, persistent, and disabling grief, that is, prolonged grief. Cognitive behavioural therapy is the first choice for prolonged grief, but it does not benefit all clients. Mindfulness‐based interventions have been proposed as an alternative treatment, yet a comprehensive review on the role of mindfulness in psychological adaptation to bereavement is lacking. Therefore, we searched PsycINFO, Web of Science and PubMed (last search: 24 February 2025; PROSPERO: CRD420251006282) to identify observational and intervention studies on the relationships of mindfulness with prolonged grief and secondary mental health problems (e.g., depression and posttraumatic stress symptoms) in bereaved adults. Thirteen studies (2097 participants) were selected. Study quality varied. Cross‐sectional (*n* = 3) and longitudinal surveys (*n* = 2) consistently showed significant associations of self‐reported mindfulness with levels of prolonged grief and secondary mental health problems. Self‐reported mindfulness also predicted changes over time in secondary mental health outcomes but not in prolonged grief symptoms. Intervention studies, including pre‐evaluations and post‐evaluations (*n* = 3), nonrandomized trials (*n* = 4) and an RCT (*n* = 1), focused mostly on secondary mental health outcomes, with only three intervention studies also including a measure of prolonged grief. Results generally supported the effectiveness of mindfulness‐based interventions for attenuating secondary mental health outcomes. Findings were mixed for prolonged grief symptoms. Altogether, findings indicated that mindfulness holds promise for improving mental health problems following bereavement. More research, including RCTs and intensive longitudinal studies, is needed to clarify the short‐ and long‐term benefits of mindfulness in people with prolonged grief.

## Summary

- Effects of self‐reported mindfulness and mindfulness‐based interventions on prolonged grief symptoms are mixed, particularly in rigorously designed longitudinal surveys and intervention studies.
- Both self‐reported mindfulness and mindfulness‐based interventions show promise in attenuating secondary mental health problems (e.g., depression and posttraumatic stress symptoms) among bereaved individuals.
- Pending future research, mindfulness‐based interventions may come to serve as a complementary or alternative treatment option for distressed bereaved adults when conventional treatments are ineffective or not preferred.

The death of a loved one can be a painful and challenging experience. While most people adjust to bereavement without the need for professional care (Bonanno and Malgaroli 2020), a small but significant minority may develop severe and persistent grief that impairs daily functioning, termed *prolonged grief*. Prolonged grief reactions may include persistent yearning and cognitive preoccupation with the deceased, a sense of disbelief about the death, a feeling as if a part of oneself has died, feelings of emotional numbness, emotional pain and intense loneliness. Diagnoses characterized by such grief responses are recognized as prolonged grief disorder (PGD) in the International Classification of Diseases eleventh edition (ICD‐11; World Health Organization 2022) and the Diagnostic and Statistical Manual of Mental Disorders‐fifth edition, text revision (DSM‐5‐TR; American Psychiatric Association 2022). Recent surveys in representative population samples estimate that 3.3%–4.2% of individuals are at risk for PGD (Rosner et al. 2021; Treml et al. 2022).

Importantly, prolonged grief symptoms are concurrently associated with and longitudinally predict a range of mental and physical health problems. These include stress‐related and affective disorders such as depression and posttraumatic stress disorder (PTSD) (Janshen and Eisma 2024; Komischke‐Konnerup et al. 2021), reduced quality of life (Eisma and Schmitt 2024), increased suicidal tendencies (Boelen and Prigerson 2007) as well as higher risks of cancer, cardiovascular disease, hypertension (Prigerson et al. 1997), bodily distress syndrome, and other chronic physical conditions (Cunningham et al. 2025).

Given its profound impact on mental and physical health problems, identifying effective interventions to help people experiencing prolonged grief is critical. When developing or selecting interventions, it is crucial to target maladaptive coping strategies such as *experiential avoidance* and *rumination*, which are risk factors for prolonged grief (for a review: Eisma and Stroebe 2021). Experiential avoidance occurs when individuals try to avoid or escape painful emotions, for instance by avoiding reminders of the death and loss‐related emotions (Chawla and Ostafin 2007). Rumination—repetitive and passive dwelling on the causes and consequences of the loss—may exacerbate emotional problems by reinforcing self‐critical thoughts and prolonging negative emotional states, and distracting people from painful loss‐related memories and emotions (Eisma et al. 2020; Eisma et al. 2013; Eisma and Stroebe 2017). While these strategies may provide temporary relief of severe loss‐related distress, they ultimately hinder adaptive emotional processing of the loss, thereby prolonging grief reactions (Eisma et al. 2013).

One type of therapy that aims to reduce prolonged grief by targeting such maladaptive coping strategies is cognitive‐behavioural therapy (CBT). CBT is the first‐choice treatment for loss‐related psychopathology (Boelen et al. 2007; Bryant et al. 2014; Lenferink et al. 2023; Rosner et al. 2014). CBT for prolonged grief may include psychoeducation about PGD, monitoring of daily thoughts and revisiting the death memory, cognitive reframing of maladaptive grief‐related thoughts, writing a letter to the deceased and activities to promote positive memories of the deceased as well as relapse prevention. Research suggests that CBT has a moderate effect in reducing prolonged grief symptoms directly post‐intervention, while large effects are observed at follow‐ups (Komischke‐Konnerup et al. 2024). However, despite its efficacy, CBT does not work for everyone; only about 50% of clients experience clinically relevant changes in prolonged grief symptoms following CBT (Doering and Eisma 2016). This highlights the need to explore alternative or complementary interventions that target putative perpetuating mechanisms of prolonged grief, particularly the use of maladaptive emotion regulation and coping strategies (Eisma and Stroebe 2021).

Mindfulness‐based interventions (MBIs) are increasingly recognized for their potential to improve emotion regulation and reduce affective and stress‐related disorder symptomatology (He et al. 2024; Li et al. 2022; Perestelo‐Perez et al. 2017). *Mindfulness* refers to the intentional awareness of being fully present in the present moment with an open, accepting and nonjudgmental attitude (Kabat‐Zinn 2003). Unlike CBT, which primarily focuses on restructuring the content of maladaptive thoughts (Lappalainen et al. 2007), MBIs encourage people to change their relationship with their thoughts and emotions by developing present‐moment awareness, acceptance and nonreactivity (Segal et al. 2018).

Mindfulness is proposed to counteract maladaptive coping processes such as avoidance and rumination by promoting exposure and acceptance (He et al. 2024; Li et al. 2022), which may allow individuals to acknowledge and experience grief without resisting and denying it. This acceptance may create psychological space for grief to be acknowledged and worked through, rather than suppressed, which enables habituation of grief‐related distress and adaptation to bereavement (Stroebe and Schut 1999). Moreover, mindfulness is theorized to break the cycle of rumination by cultivating meta‐awareness, which enables individuals to observe their thoughts without being overwhelmed by them (also referred to as ‘decentering’) (Huang 2022). Instead of being caught in a cycle of intrusive thoughts, regret and (self) blame, individuals practicing mindfulness can develop a more balanced and compassionate perspective toward themselves and the loss (Scocco et al. 2022). Neuroscientific research supports these effects by showing that mindfulness practice increases prefrontal cortex activity, which is implicated in emotion regulation (Tang et al. 2015).

Some empirical evidence supports these theorized benefits of mindfulness in adaptation to bereavement, with observational studies supporting negative concurrent associations of self‐reported mindfulness with prolonged grief and depression symptoms (e.g., Eisma et al. 2023; Huang 2022; Tang et al. 2019). However, some inconsistent findings have also been reported in the literature. For example, a controlled nonrandomized pilot trial found that mindfulness‐based cognitive therapy (MBCT) significantly reduced depression symptoms but not prolonged grief symptoms in older bereaved adults (O'Connor et al. 2014).

## 1 The Current Study

Despite the clinical relevance and growing scientific interest in mindfulness in adjustment to bereavement, and mixed findings in the empirical literature, a systematic review on the topic is lacking. Therefore, the goal of this review is to summarize existing quantitative empirical evidence on the relationship of self‐reported mindfulness with prolonged grief symptoms and secondary mental health problems (e.g., general grief severity, depressive, anxiety and posttraumatic stress symptoms) and on the effects of MBIs on prolonged grief symptoms and these secondary mental health outcomes. By integrating findings from observational and intervention studies, this review aims to clarify the potential role of mindfulness in addressing loss‐related psychopathology, while simultaneously identifying gaps in the current literature and avenues for future research, with the ultimate aim of improving clinical practice for severely distressed bereaved adults.

## 1 Method

### 1.1 Preregistration

This systematic review was preregistered in PROSPERO's international prospective register of systematic reviews (registration number: CRD420251006282), available at: https://www.crd.york.ac.uk/PROSPERO/view/CRD420251006282.

### 1.2 Search Strategy and Study Selection

The systematic searches were conducted in PsycINFO, Web of Science and PubMed databases using the following keywords (All Fields): ‘grief’ OR ‘bereav*’ OR ‘mourn*’ AND ‘mindfulness’. The search was limited to empirical studies with no restriction on publication dates. The final search was performed on 24 February 2025 and yielded 269 potentially relevant studies. After removing 107 duplicates, 162 studies remained for screening.

**FIGURE 1:**
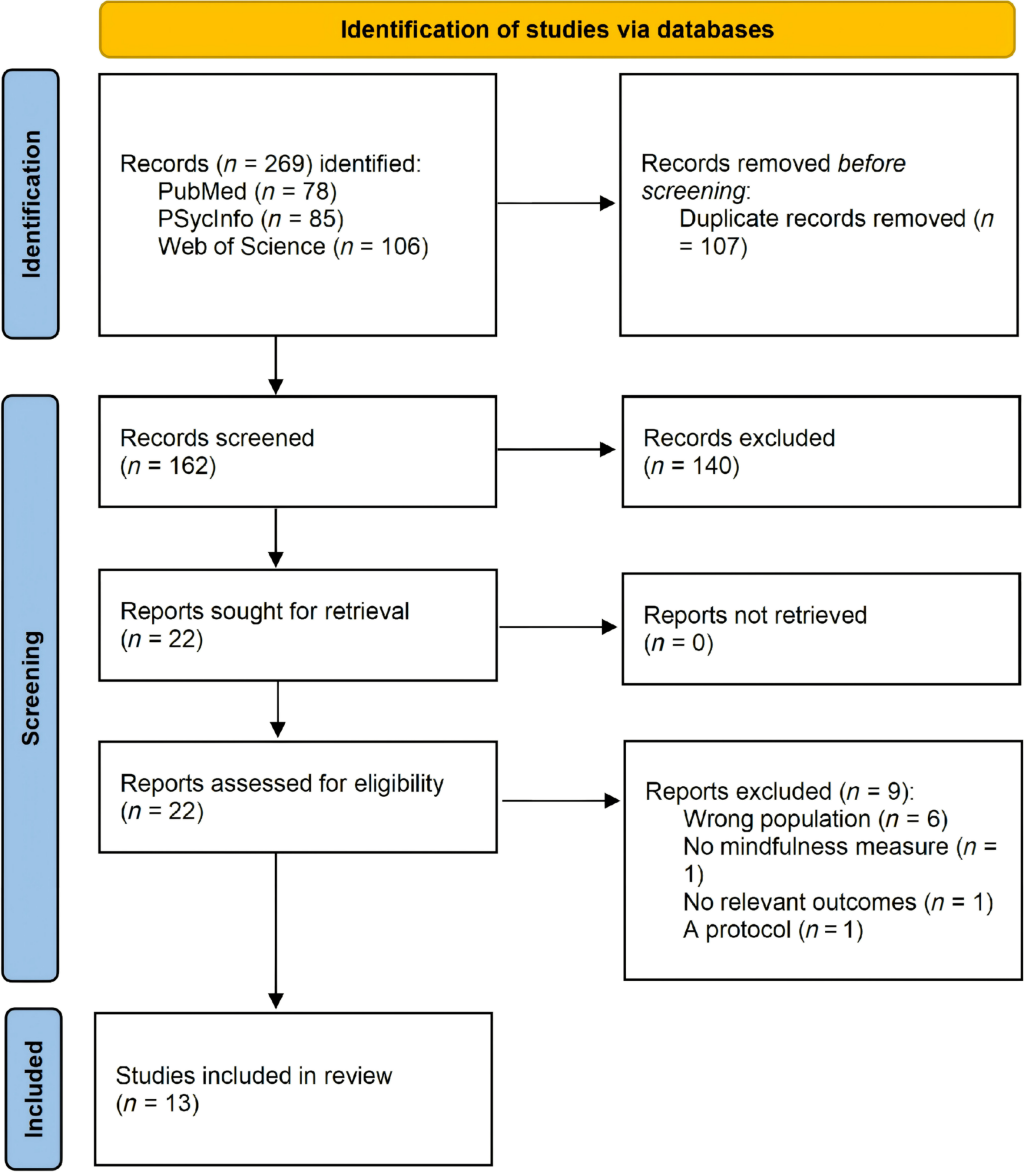
PRISMA flow diagram.

The screening and selection process was conducted by two reviewers (XS and ML) independently using Covidence software. Discrepancies were resolved through discussion until mutual consensus was achieved. Following title and abstract screening, 22 studies were selected for full‐text review. After reviewing the full texts by the two reviewers independently, 13 studies met the inclusion criteria for the systematic review. No additional studies were identified after reviewing the reference lists of the 13 included studies. The study selection process is summarized in a PRISMA flowchart (see Figure 1).

### 1.3 Inclusion and Exclusion Criteria

To safeguard methodological quality and interpretability, only quantitative, peer‐reviewed studies published in English were eligible for inclusion. Furthermore, a minimum sample size of *N* = 20 was set to reliably detect large effect sizes (*r* ≥ 0.80; Cohen 1988) with 0.80 power. Eligible studies were restricted to adult participants (≥ 18 years) who had experienced the death of another human being. Observational studies (cross‐sectional and longitudinal) were included if they used at least one standardized quantitative measure of mindfulness, along with at least one standardized quantitative measure of prolonged grief symptoms or a secondary bereavement‐related mental health outcome (e.g., general grief severity, depression, anxiety or posttraumatic stress symptoms). Interventions were included if they explicitly evaluated the efficacy of MBIs targeting prolonged grief symptoms or secondary bereavement‐related mental health outcomes. To ensure that observed effects could be attributed to mindfulness, we further required interventions to have mindfulness as the primary component, rather than being part of broader multicomponent interventions where mindfulness is only a minor element (e.g., Sveen et al. 2025).

Studies were excluded if they exclusively examined associations between mindfulness and positive psychological outcomes (e.g., posttraumatic growth) or coping styles (e.g., rumination) as our review focused specifically on mental health problems related to bereavement. Additionally, studies were excluded if they focused exclusively on grief related to stillbirth, given that grief following stillbirth may be qualitatively distinct from other loss types (Brier 2008). Study protocols and studies without empirical data were also excluded.

### 1.4 Quality Assessment

Two reviewers (XS and ML) independently assessed the methodological quality of the included studies using the Mixed Methods Appraisal Tool (MMAT, version 2018; Hong et al. 2018), a validated and reliable tool designed to evaluate different study designs (Hong et al. 2019; Souto et al. 2015). Following MMAT (2018) categories, the studies included were classified as follows: Cross‐sectional and longitudinal observational studies without exposure variables (i.e., MBIs) were evaluated as quantitative descriptive studies; pre‐evaluation and postevaluation studies and nonrandomized trials with exposure/intervention but no randomization were evaluated as quantitative nonrandomized studies; the one RCT was evaluated as such.

According to MMAT, each study was first screened using two preliminary questions (see Table 1). Following screening, included studies that met these screening criteria were evaluated based on MMAT criteria corresponding to their study design categories. Assessment responses were rated as ‘Yes’, ‘No’ or ‘Cannot tell’. The two reviewers compared their ratings and resolved any discrepancies through discussion. The initial interrater reliability, calculated using Cohen's kappa based on the five MMAT quality assessment criteria, was 0.76, indicating substantial agreement. The overall initial agreement across these quality items was 88%.

**TABLE 1:** Quality assessment.

Quantitative descriptive studies	Is the sampling strategy relevant to address the research question?	Is the sample representative of the target population?	Are the measurements appropriate?	Is the risk of nonresponse bias low?	Is the statistical analysis appropriate to answer the research question?
Huang (2022)	Yes	No	Yes	Cannot tell	Yes
Tang et al. (2019)	Yes	No	Yes	Yes	Yes
Wang et al. (2024)	Yes	No	Yes	No	Yes
Eisma et al. (2023)	Yes	No	Yes	Yes	Yes
Emrich et al. (2023)	Yes	No	Yes	Yes	Yes

Quantitative nonrandomized studies	Are the participants representative of the target population?	Are measurements appropriate regarding both the outcome and intervention (or exposure)?	Are there complete outcome data?	Are the confounders accounted for in the design and analysis?	During the study period, is the intervention administered (or exposure occurred) as intended?
Huang et al. (2019)	No	Yes	Yes	Cannot tell	Yes
Thieleman and Cacciatore (2014)	No	Yes	Yes	No	Cannot tell
Scocco et al. (2019)	No	Yes	Yes	Cannot tell	Yes
Knowles et al. (2021)	No	Yes	Yes	Yes	Yes
O'Connor et al. (2014)	No	Yes	No	Yes	Yes
Scocco et al. (2022)	No	Yes	Yes	Yes	Cannot tell
Thieleman and Cacciatore (2020)	No	Yes	No	No	Yes

Quantitative randomized controlled trials	Is randomization appropriately performed?	Are the groups comparable at baseline?	Are there complete outcome data?	Are outcome assessors blinded to the intervention provided?	Did the participants adhere to the assigned intervention?
Bryant et al. (2024)	Yes	Yes	No	Yes	Yes

*Note:* Two screening questions: (1) Are there clear research questions? (2) Do the collected data allow for addressing the research questions? Only studies that met both criteria were included in the full MMAT quality appraisal.

### 1.5 Data Extraction and Synthesis

Two reviewers (XS and ML) independently extracted the following data from each included study: first author, publication year, country of first author's affiliation, study design (cross‐sectional survey, longitudinal survey, pre–post study, nonrandomized controlled trial, RCT), sample size, demographic characteristics (i.e., age and gender), loss‐related characteristics (i.e., time since loss, relationship with the deceased and cause of death), MBI and details (i.e., types and duration of intervention), characteristics of the mindfulness measure and measures assessing prolonged grief symptoms or related mental health outcomes (i.e., measure name, reference, number of items and response scale), statistical results and effect sizes concerning the relations between mindfulness and prolonged grief symptoms or other bereavement‐related mental health outcomes. Any discrepancies between the authors during data extraction were resolved through discussion until consensus was reached. Following extraction, a senior author (ME) double‐checked all extracted information. Due to methodological heterogeneity, a meta‐analysis was not feasible. Extracted data are summarized in Table 2. More detailed information on the mindfulness interventions, including intervention names, session durations and grief‐specific adaptations, is provided in Table S1.

**TABLE 2:** Summary of main findings on mindfulness and bereavement‐related mental health symptoms.

Study (first author, year)	*N*	Sample characteristic at baseline	Inclusion criterion (population type)	Study design	Mindfulness measure(s)/intervention(s)	Prolonged grief and other bereavement‐related symptom measures	Main relevant findings
Huang (2022)	509	Taiwan, 63% female, M_age_ = 45.98 years, time since loss: within the previous 3 years, deceased is: parent (38%), child (10%), spouse (13%), sibling (11%), grandparent (29%), cause of death: not specified	—	Cross‐sectional Survey	FFMQ (Baer et al. 2008), 39 items/No intervention	PG measure: ICG (Prigerson et al. 1995), 19 items Secondary mental health measures: Taiwanese Depression Scale (Lee et al. 2000), 18 items; TRIG (Faschingbauer et al. 1977), 21 items (8 for past grief, 13 for present grief)	Mindfulness correlated significantly negatively with PG (*r* = −0.37), past general grief severity (*r* = −0.38), present general grief severity (*r* = −0.23) and depression symptoms (*r* = −0.50).
Tang et al. (2019)	393	China, 56% female, M_age_ = 31.94 years, M time since loss: 16.88 months, deceased is: spouse (11%), child (10%), parent (34%), sibling (46%), cause of death: chronic illness (22%), acute illness (38%), accident (39%), suicide (1%)	—	Cross‐sectional Survey	MAAS (Brown and Ryan 2003), Chinese version (Deng et al. 2012), 15 items/no intervention	PG measure: PGQ (Prigerson et al. 2009), Chinese version (He et al. 2013), 11 items; Secondary mental health measure: HADS (Zigmond and Snaith 1983), Chinese version (Ye and Xu 1993), 14 items	In hierarchical regression analyses, including loss‐related variables and grief rumination, higher self‐reported mindfulness was significantly associated with lower PG (*β* = −0.18), depression (*β* = −0.25) and anxiety (*β* = −0.31).
Wang et al. (2024)	111	China (sample location; first author's affiliation: Japan), 61% female, M_age_ = 64.85 years, M time since loss: 178.8 months, deceased is: child (100%), cause of death: not specified	—	Cross‐sectional Survey	MAAS (Brown and Ryan 2003), Chinese version (Chen et al. 2023), 15 items/no intervention	PG measure: no Secondary mental health measure: GDS‐15 (Brink et al. 1982), 15 items	Mindfulness and depressive symptoms were significantly negatively correlated (*r* = −0.70).
Eisma et al. (2023)	397	Netherlands, 89% female, M_age_ = 53.68 years, M time since loss = 18.02 months, deceased is: partner (48%), parent (31%), sibling (5%), child (12%), other (4%), cause of death: nonviolent (87%), violent (13%)	—	Longitudinal Survey	MAAS (Brown and Ryan 2003); Dutch version (Schroevers et al. 2008), 6 items/no intervention	PG measure: TGI‐SR+ (Lenferink et al. 2022), 22 items Secondary mental health measure: QIDS (Rush et al. 2003), Dutch version (Lako et al. 2014), 16 items	Baseline mindfulness was significantly correlated with PG symptoms both at baseline (*r* = −0.53) and at 6‐month follow‐up (*r* = −0.49), as well as with depressive symptoms at baseline (*r* = −0.60) and at 6‐month follow‐up (*r* = −0.53). In hierarchical regression analyses, controlling for background variables, cognitive reappraisal and emotional expression, baseline mindfulness significantly predicted lower baseline PG symptoms (*β* = −0.43) and depressive symptoms (*β* = −0.55). At follow‐up, baseline mindfulness significantly predicted depressive symptoms (*β* = −0.11), but not PG symptoms (*β* = −0.07), after controlling for baseline symptom levels.
Emrich et al. (2023)	117	USA, 79% female, M_age_ not specified, time since loss: 0–3 months (23%), 4–6 months (20%); 7–12 months (30%); > 1 year ago (27%), deceased is: grandparent (42%), other family member (21%), parent/caregiver (15%), nonromantic friend (15%), romantic partner (1%), other close relationship (7%), cause of death: prolonged illness (28%), sudden illness (25%), accident (12%), natural/old age (11%), suicide (8%), homicide (3%), other (14%)	—	Longitudinal Survey	CAMS‐R (Feldman et al. 2007), 10 items/no intervention	PG measure: no Secondary mental health measures: DASS‐21 (Lovibond and Lovibond 1995), 21 items PROMIS Sleep Disturbance Short Form (Yu et al. 2012), 8 items	Mindfulness at baseline significantly correlated negatively with sleep disturbances 3 weeks later (*r* = −0.43) and with depression (*r* = −0.53), anxiety (*r* = −0.42) and stress levels (*r* = −0.50) 6 weeks later. In a mediation model, controlling for baseline levels of depression, anxiety, stress, and positive states of mind, baseline mindfulness significantly predicted lower levels of depression (*β* = −0.46), anxiety (*β* = −0.34) and stress (*β* = −0.51) 6 weeks later, with sleep disturbances included as a mediating variable.
Huang et al. (2019)	23	Taiwan, 91% female, M_age_ = 48.35 years, time since loss: 6 months to 4 years, deceased is: not specified, cause of death: not specified	Loss within 6 months–4 years; self‐reported unresolved grief required (indicated)	Pre‐evaluation and postevaluation study	FFMQ (Baer et al. 2008), 39 items/MBCT: 8 weeks (2.5 h/week + home practice), plus 2‐h grief‐specific introduction	PG measure: no Secondary mental health measures: TRIG (Faschingbauer et al. 1987), (only present grief part used, 13 items); GAD‐7 (Spitzer et al. 2006), 7 items; Taiwanese Depression Scale (Lee et al. 2000), 18 items	Receiving MBCT coincided with significant reductions in general grief severity (*d* = −0.89), depression (*d* = −1.17) and anxiety (*d* = −0.65).
Thieleman et al. (2014)	42	USA, 71% female, M_age_ = 38.98 years, M time since loss: 20.52 months, deceased is: child (81%), rest is not mentioned, cause of death: not specified	Traumatic‐bereavement sample; self‐referred for counselling; no clinical cutoff required (selective)	Pre‐evaluation and postevaluation study	No measure/Mindfulness‐based bereavement Care (ATTEND), with average total of 14.64 h of individual counselling	PG measure: no Secondary mental health measures: IES‐R (Weiss and Marmar 1997), 22 items, 3 subscales HSCL‐25 (Veijola et al. 2003), 25 items, 2 subscales	Receiving the mindfulness intervention coincided with a statistically significant decline in symptoms of depression and anxiety (*d* = −0.70) as well as posttraumatic stress (*d* = −0.92) from pre‐test to post‐test.
Scocco et al. (2019)	61	Italy, 80% female, M_age_ = 49.5 years, M time since loss: 27.61 months, deceased is: son/daughter (34%), father/mother (5%), brother/sister (20%), spouse/partner (33%), other close relative (8%), cause of death: suicide (100%)	Suicide‐bereaved adults and at least 3 months post‐loss (selective)	Pre‐evaluation and postevaluation study	FFMQ (Baer et al. 2006), 39 items; Mindfulness subscale of SCS (Neff 2003b, 2003a), 26 items/Mindfulness‐based weekend retreats (*Panta Rhei*), based on MBSR, MBCT and MSC, 16 h over a weekend	PG measure: no Secondary mental health measure: POMS (McNair et al. 1971), 58 items, 6 subscales	Participation in *Panta Rhei* coincided with significant decreases in tension–anxiety, depression–dejection, anger–hostility, fatigue–inertia, and confusion–bewilderment from pre‐ to post‐intervention. Vigour–activity did not significantly change. Effect sizes not reported.
Knowles et al. (2021)	95	USA, 79% female, M_age_ = 67.45 years, M time since loss = 14.67 months, deceased is: spouse/romantic partner (100%), cause of death: not specified	Bereaved spouses/partners, 6–48 months post‐loss (selective)	Nonrandomized controlled trial	EQ‐D (Fresco et al. 2007), 11 items/Waitlist vs. PMR 6 weeks vs. MT 6 weeks (2‐h weekly sessions)	PG measure: Revised ICG (Prigerson and Jacobs 2001), 17 items Secondary mental health measure: CES‐D (Radloff 1977), 20 items	The overall group × time interaction on PG symptoms was not statistically significant controlling for baseline depression levels. However, the PMR group showed a significantly greater reduction in PG symptoms compared with the waitlist at the 1‐month follow‐up (*d* = 0.47). No significant differences in PG symptom changes were observed between the MT and waitlist, nor between the MT and PMR groups. Changes in depression symptoms were not reported.
O'Connor et al. (2014)	36	Denmark, 70% female, M_age_ = 76.85 years, time since loss: approximately 4 years post‐loss, deceased is: spouse (100%), cause of death: not specified	Spouse‐loss adults (65–80 years), ≥ 4 years post‐loss; participants with elevated levels of psychological distress related to the death of the spouse (post‐traumatic stress, depression, or prolonged grief [indicated])	Nonrandomized controlled trial	No measure/Wait list control group vs. MBCT: 8 weeks (2‐h sessions) + 2 booster sessions at 3‐ and 6‐months post‐intervention	PG measure: Revised ICG (Jacobs et al. 2000 Prigerson et al. 1995), 15 items Secondary mental health measure: BDI‐2 (Beck et al. 1988), 21 items; HTQ (Mollica et al. 1992), 16 items	Completers analyses demonstrated a significant time × group interaction effect from pretest to 5‐month follow‐up on depressive symptoms (*g* = 0.88), indicating that MBCT led to greater reductions in depression than the waitlist condition at follow‐up, although no significant group differences were observed immediately post‐intervention. However, the intention‐to‐treat (ITT) analysis revealed a nonsignificant time × group interaction from pretest to 5‐month follow‐up (g = 0.49). Completers and ITT analyses showed no significant time × group interaction effects on posttraumatic stress (*g*'s = 0.24 and 0.12, respectively) and PG symptoms (*g*'s = 0.02 and 0.05, respectively).
Scocco (2022)	147	Italy, 82% female, M_age_ = 47.62 years, time since loss: 0–6 months (11%), 6–12 months (24%), 1–2 years (27%), > 2 years (30%), deceased is: son/daughter (31%), father/mother (13%), brother/sister (19%), spouse/partner (29%), other loved one (8%), cause of death: suicide (100%)	Suicide‐bereaved adults and at least 3 months post‐loss (selective)	Nonrandomized controlled trial	FFMQ (Baer et al. 2006), 39 items Mindfulness subscale of SCS (Neff 2003b), 26 items/control group vs. *Panta Rhei*: short, intensive, mindful self‐compassion‐based intervention, based on MBSR, MBCT and MSC: 16 h over a weekend	PG measure: no Secondary mental health measure: POMS (McNair et al. 1971), 58 items, 6 subscales	Significant group × time interactions were found, indicating that the intervention group experienced greater reductions in total psychological distress, tension–anxiety, depression–dejection, anger–hostility, fatigue–inertia, and confusion–bewildermen, as well as a significant increase in vigour‐activity, compared with the control group. Effect sizes were not reported.
Thieleman (2020)	66	USA, 94% female, M_age_ = 42.02 years, M time since loss: 50.88 months, deceased is: child (100%), cause of death: unknown (24%), illness/anomaly (45%), accident/suicide/homicide (30%)	Parents bereaved of a child (selective)	Nonrandomized controlled trial	FFMQ (Baer et al. 2006), 39 items (only Describing, Acting with Awareness, Nonjudging of Inner Experience, and Nonreactivity to Inner Experience were reported)/Online support forum vs. Selah: Contemplative retreat for traumatic bereavement for 4 days	PG measure: no Secondary mental health measures: IES‐R, 22 items; HSCL‐25 (Veijola et al. 2003), 25 items	Significant time × condition interactions were found for depression (*η* ^2^_*p*_ = 0.10) and posttraumatic stress symptoms (*η* ^2^_*p*_ = 0.12), but not for anxiety, indicating that the intervention group experienced greater symptom reductions over time compared with the online support forum group. Post hoc comparisons showed that at post‐intervention, the intervention group reported significantly lower levels of depression (*η* ^2^_*p*_ = 0.07) and posttraumatic stress (*η* ^2^_*p*_ = 0.11) than the comparison group; however, these between‐group differences were no longer significant at the 5‐ to 9.5‐week follow‐up.
Bryant et al. (2024)	100	Australia, 87% female, M_age_ = 47.3 years, M time since loss = 44.8 months, deceased is: parent (35%), sibling (11%), partner (23%), child (29%), grandparent (2%), cause of death: medical fatality (55%), homicide (2%), unintentional injury (20%), suicide (23%)	Adults meeting ICD‐11 criteria for prolonged grief disorder (indicated)	Randomized Controlled Trial	No measure/Grief‐focused CBT vs. MBCT (11 weekly 90‐min individual sessions)	PG measure: PG‐13 (Prigerson et al. 2009), 13 items Secondary mental health measures: BDI‐2 (Beck et al. 1996), 21 items BAI (Steer and Beck 1997), 21 items	No differences between GF‐CBT and MBCT at post‐treatment on PG, depression, and anxiety symptoms, but GF‐CBT was more efficacious than MBCT at 6‐month follow‐up in reducing PG (*d* = 0.8) and depression symptoms (*d* = 0.6), not anxiety.

*Note:* Based on the inclusion criteria, interventions were classified as follows: Universal, targeting all bereaved individuals regardless of risk level or symptom severity; Selective, targeting only higher‐risk grievers such as parents who lost children; Indicated, targeting those assessed to have difficulty adapting to the loss (e.g., high levels of prolonged grief).

Abbreviations: BAI: Beck Anxiety Inventory; BDI‐2: Beck Depression Inventory‐2; CAMS‐R: Cognitive Affective Mindfulness Scale‐Revised; CES‐D: Center for Epidemiological Studies Depression Scale; DASS‐21: Depression Anxiety Stress Scales; EQ‐D: Experiences Questionnaire Decentering subscale; FFMQ: Five‐Facet Mindfulness Questionnaire; GAD‐7: Generalized Anxiety Disorder‐7; GDS‐15: Geriatric Depression Scale; HADS: Hospital Anxiety and Depression Scale; HSCL‐25: Hopkins Symptom Checklist‐25; HTQ: Harvard Trauma Questionnaire—Part IV; ICG: Inventory of Complicated Grief; ICD‐11: International Classification of Diseases, 11th Revision; IES‐R: Impact of Event Scale–Revised; MAAS: Mindful Attention Awareness Scale; MBCT: Mindfulness‐Based Cognitive Therapy; MBSR: Mindfulness‐Based Stress Reduction; MSC: Mindful‐Self‐Compassion; MT: mindfulness training; PG‐13: Prolonged Grief‐13 scale; PG: Prolonged Grief; PGQ: Prolonged Grief Questionnaire; PMR: progressive muscle relaxation; POMS: Profiles of Mood States; QIDS: Quick Inventory of Depressive Symptomatology; SCS: Self‐Compassion Scale; TGI‐SR+: Traumatic Grief Inventory‐Self Report Plus; TRIG: Texas Revised Inventory of Grief.

## 2 Results

### 2.1 Study Characteristics

The 13 eligible studies collectively included 2097 bereaved participants. Three included studies were cross‐sectional surveys (23%), two were longitudinal surveys (15%), three were pre‐evaluation and postevaluation studies (23%), four were nonrandomized controlled trials (31%), of which one included randomized and nonrandomized groups (Knowles et al. 2021), and one was a randomized controlled trial (8%).

To summarize the main sample characteristics across studies (weighted by sample size): participants had a mean age of 47.43 years (SD = 15.80), and, on average, 73% were female. Forty‐six per cent of the studies (*n* = 6) included bereaved individuals with varying kinship relations to the deceased. Among the studies focusing on specific relationships, the most frequently studied types of bereavement were the loss of a child (23%, *n* = 3) and the loss of a spouse (23%, *n* = 3). One study did not specify the type of relationship with the deceased. Across nine studies that reported a cause of death, a majority of participants had experienced a natural death (58%) and a minority an unnatural death (40%). An additional 2% were categorized as ‘other’. The average time since loss at baseline was 35.90 months (SD = 57.35).

### 2.2 Study Quality

The methodological quality of the 13 included studies is summarized in Table 1. As the use of scoring is not recommended in the 2018 MMAT guidelines, detailed quality appraisal information is presented.

All 13 included studies met the two initial MMAT screening criteria and were subsequently evaluated according to design‐specific criteria. The five quantitative descriptive studies generally used relevant sampling strategies, appropriate measurements and employed adequate statistical analyses. However, none of these studies included a representative population sample. Additionally, for some studies, there were concerns regarding nonresponse bias or unclear reporting (Huang 2022; Wang et al. 2024). Among the seven quantitative nonrandomized studies (Huang et al. 2019; Knowles et al. 2021; O'Connor et al. 2014; Scocco et al. 2022; Scocco et al. 2019; Thieleman and Cacciatore 2020; Thieleman et al. 2014), all used appropriate outcome and intervention measurements, and most ensured complete outcome data and proper administration of the intervention (Huang et al. 2019; Knowles et al. 2021; Scocco et al. 2022, 2019; Thieleman et al. 2014). However, participants were not representative of the target population, and some studies lacked sufficient control for confounders or did not clearly report on confounding (Huang et al. 2019; Scocco et al. 2019; Thieleman and Cacciatore 2020; Thieleman et al. 2014). The only RCT (Bryant et al. 2024) met four out of five quality criteria, with clearly described randomization procedures, assessor blinding and good adherence to assigned interventions, but dropout resulted in concerns about incomplete outcome data.

### 2.3 Main Findings

Detailed information on the sample characteristics, measures and main findings from the studies is presented in Table 2.

#### 2.3.1 Observational Studies

A total of five observational studies (three cross‐sectional, two longitudinal) examined associations between self‐reported mindfulness and bereavement‐related mental health problems.

The cross‐sectional studies showed that higher levels of self‐reported mindfulness were significantly associated with lower levels of prolonged grief (Huang 2022; Tang et al. 2019), general grief severity (Huang 2022), depression (Huang 2022; Tang et al. 2019; Wang et al. 2024) and anxiety (Tang et al. 2019).

Two longitudinal studies yielded mixed findings. A two‐wave longitudinal survey demonstrated that higher baseline mindfulness related to lower concurrent and future prolonged grief and depression symptoms using zero‐order correlations. Additionally, mindfulness predicted decreased depressive symptoms, but not prolonged grief symptoms over 6 months, whilst controlling for baseline symptoms (Eisma et al. 2023). In a three‐wave longitudinal study, Emrich et al. (2023) demonstrated that higher baseline mindfulness was negatively correlated with sleep disturbances at 3 weeks and with stress, anxiety and depression levels at 6 weeks. Additionally, in three separate predictive models controlling for baseline levels of dependent variables (levels of depression, anxiety, stress and positive states of mind), baseline mindfulness significantly predicted lower levels of depression, anxiety and stress 6 weeks later.

#### 2.3.2 Intervention Studies

Eight intervention studies (three pre‐evaluation and postevaluation, four nonrandomized controlled trials and one randomized controlled trial) examined the effects of MBIs on bereavement‐related mental health problems.

Three pre‐evaluation and postevaluation studies assessed whether receiving MBIs coincided with improvements in mental health. The duration and format of these interventions varied, including a 2‐day mindfulness retreat (Scocco et al. 2019), an 8‐week MBCT course (Huang et al. 2019) and an average of 14.64 h of individual counselling (Thieleman et al. 2014). No pre–post studies assessed changes in prolonged grief symptoms. These studies consistently reported significant improvements in secondary mental health outcomes after receiving MBI, including general grief severity (Huang et al. 2019), depression (Huang et al. 2019; Thieleman et al. 2014), anxiety (Huang et al. 2019; Thieleman et al. 2014), posttraumatic stress symptoms (Thieleman et al. 2014) and psychological distress (Scocco et al. 2019).

Four nonrandomized controlled trials compared MBIs to waitlist or active controls. In two of the four trials, the effects of MBI on prolonged grief were examined, along with secondary mental health outcomes (Knowles et al. 2021; O'Connor et al. 2014); the other two trials focused only on secondary mental health outcomes (Scocco et al. 2022; Thieleman and Cacciatore 2020). No significant effects were found for prolonged grief symptoms (Knowles et al. 2021; O'Connor et al. 2014). Significant beneficial effects were found on several secondary mental health outcomes, including reductions in depression symptoms (O'Connor et al. 2014; Thieleman and Cacciatore 2020), posttraumatic stress symptoms (Thieleman and Cacciatore 2020) and psychological distress (Scocco et al. 2022).

Specifically, O'Connor et al. (2014) showed in a completers analysis that 8‐week MBCT significantly reduced depressive symptoms relative to a waitlist group at the 5‐month follow‐up, although no significant group differences were observed at post‐test. This effect was not replicated in the intention‐to‐treat analysis. No significant group × time interactions emerged for prolonged grief or posttraumatic stress symptoms. Thieleman and Cacciatore (2020) reported that a 4‐day mindfulness‐based retreat (vs. online support forum) led to greater reductions in depression and posttraumatic stress symptoms at post‐test—but not at the 5‐ to 9.5‐week follow‐up. No significant effects were found for anxiety. Knowles et al. (2021) evaluated the effects of mindfulness training, progressive muscle relaxation and a waitlist control on prolonged grief symptoms. No significant overall group × time interaction effects were found. However, the progressive muscle relaxation group reported significantly lower prolonged grief symptoms compared with the waitlist group at follow‐up, whereas no significant differences were found between the mindfulness training group and the other two groups. Scocco et al. (2022) reported that, compared with a waitlist control group, a weekend mindfulness retreat led to significant improvements in psychological distress, including reductions in tension–anxiety, depression–dejection, anger–hostility, fatigue–inertia and confusion–bewilderment, as well as a significant increase in vigour–activity.

The only (fully) randomized controlled trial (Bryant et al. 2024) compared MBCT with grief‐focused CBT. Both interventions were eleven 90‐min sessions. Immediately post‐treatment, no significant differences were found between the two interventions on prolonged grief, depression and anxiety symptoms. However, at the 6‐month follow‐up, grief‐focused CBT was more effective than MBCT in reducing prolonged grief and depression symptoms, while no significant differences were found for anxiety symptoms.

## 3 Discussion

This systematic review synthesized existing evidence on the relationships of self‐reported mindfulness with prolonged grief symptoms and secondary mental health outcomes, such as general grief severity, depression, anxiety and posttraumatic stress symptoms. In addition, we summarized the effects of MBIs on prolonged grief symptoms and secondary mental health outcomes. We selected 13 studies, collectively including 2097 bereaved participants: three cross‐sectional and two longitudinal surveys, as well as three preintervention and postintervention studies, four nonrandomized controlled trials and one randomized controlled trial.

Cross‐sectional observational studies provided consistent support for inverse relationships of mindfulness with prolonged grief symptoms (Huang 2022; Tang et al. 2019), general grief severity (Huang 2022), depressive symptoms (Huang 2022; Tang et al. 2019; Wang et al. 2024) and anxiety symptoms (Tang et al. 2019). These findings are in line with evidence from other systematic reviews showing the negative associations of self‐reported mindfulness with mental health problems (for reviews: Eisma and Stroebe 2021; Naragon‐Gainey et al. 2017). The two longitudinal studies painted a more nuanced picture. Controlling for baseline symptoms, mindfulness predicted reductions in secondary mental health outcomes such as depression symptoms (Eisma et al. 2023; Emrich et al. 2023), anxiety symptoms and levels of stress and sleep disturbances (Emrich et al. 2023) but not in prolonged grief symptoms (Eisma et al. 2023).

Intervention studies showed a complex picture. As pre‐evaluation and postevaluation studies did not assess changes in prolonged grief symptoms, no conclusions can be drawn from these studies about the potential efficacy of MBI on prolonged grief symptoms. Results did show that receiving an MBI consistently coincided with significant improvements in general grief severity (Huang et al. 2019), as well as in levels of depression (Huang et al. 2019; Thieleman et al. 2014), anxiety (Huang et al. 2019; Thieleman et al. 2014), posttraumatic stress (Thieleman et al. 2014) and psychological distress (Scocco et al. 2019). These results suggest potential short‐term benefits of MBIs for bereaved adults. However, the absence of a control group precludes causal inferences, as symptom reduction may also be due to the natural recovery processes or expectancy effects.

Two of the four nonrandomized controlled trials included a measure of prolonged grief, showing no significant effects of MBIs relative to control groups for prolonged grief symptoms (Knowles et al. 2021; O'Connor et al. 2014). Some showed beneficial effects on secondary bereavement‐related mental health outcomes, including depression symptoms (O'Connor et al. 2014; Thieleman and Cacciatore 2020), posttraumatic stress symptoms (Thieleman and Cacciatore 2020) and psychological distress (Scocco et al. 2022). The one randomized controlled trial in our review found that both grief‐focused CBT and MBCT coincided with reductions of prolonged grief, depression and anxiety symptoms from pre‐test to post‐test (Bryant et al. 2024). At 6‐month follow‐up, grief‐focused CBT was more effective than MBCT in maintaining improvements in prolonged grief and depression symptoms, but not anxiety symptoms. While this suggests that CBT is more effective than MBCT in reducing bereavement‐related mental health problems in the long run, strong reductions in mental health problems were observed over time in both groups. The lack of a passive control condition in this randomized controlled trial leaves questions about the specific effects of MBCT on mental health unresolved. Also, CBT contained more specific techniques to target PGD than the MBCT intervention. This may have affected the outcomes. Notably, a recent secondary analysis of a controlled trial (published outside of the timeframe for our review) demonstrated that a MBI significantly reduced depression and stress levels among bereaved individuals relative to a waitlist (Knowles et al. 2025), whereas the controlled trial found no significant improvements in prolonged grief symptoms (Knowles et al. 2021).

It can be surmised that the beneficial effects of self‐reported mindfulness and MBIs appear more promising for secondary mental health outcomes than for prolonged grief. However, it should be taken into account here that prolonged grief was not measured in a significant number of the included studies, with most studies focusing on a range of secondary mental health outcomes. The benefits of mindfulness identified in our review are in line with theoretical frameworks that conceptualize mindfulness as a transdiagnostic protective factor across a wide range of psychological conditions (Alsubaie et al. 2017). Building on this framework, empirical research indicates that mindfulness may reduce distress by disrupting maladaptive cognitive and emotional processes like rumination and experiential avoidance (He et al. 2024; Mao et al. 2023), which are known to contribute to emotional distress following loss (Eisma et al. 2022; Eisma et al. 2013, 2020).

However, it remains to be established why the long‐term predictive effects of self‐reported mindfulness and the effects of MBIs on prolonged grief were less convincing. Multiple explanations for this discrepancy are possible. First, although most of the MBIs included in this review reported some form of adaptation to grief (see Table S1), these adaptations were varied and relatively general, such as providing a grief‐related rationale in the introductory session (e.g., Knowles et al. 2021) or including brief grief‐focused meditation practices (e.g., Scocco et al. 2019). Such minor adaptations may not be sufficient to address the unique symptomatology of prolonged grief, including intense yearning, emotional pain and identity confusion (Killikelly et al. 2025; Kristensen et al. 2017). Moreover, in the rare cases where mindfulness treatments were tailored more extensively, the active elements may not have changed all underlying processes that play a role in the perpetuation of prolonged grief (Bryant et al. 2024). In contrast, the first‐choice treatment for PGD, grief‐focused CBT, explicitly incorporates multiple strategies aimed at changing processes that are proposed to perpetuate prolonged grief symptoms, such as avoidance of loss‐related memories through exposure and maladaptive beliefs through cognitive restructuring (Boelen et al. 2007; Bryant et al. 2024).

Second, the populations targeted in the intervention studies may account for the observed effects. Based on inclusion criteria, interventions aiming to reduce grief severity can be classified as universal, selective or indicated interventions (Gordon 1983). Universal interventions (targeting anyone who has suffered bereavement) generally failed to produce statistically significant effects, while selective interventions (targeting higher risk individuals such as bereaved parents) showed small effects at post‐treatment, which were not maintained at follow‐up. In contrast, interventions targeting individuals with elevated levels of bereavement‐related mental health problems (e.g., prolonged grief) demonstrated more enduring effects at both post‐treatment and follow‐up (Currier et al. 2008). In our review, the strongest evidence that mindfulness reduced prolonged grief symptoms came from an indicated intervention involving participants meeting diagnostic criteria for PGD (Bryant et al. 2024). This focus may partly explain the strong observed within‐group effects in this trial.

Third, differences in mindfulness facets may also play a role in explaining the findings (Baer et al. 2006). According to the Monitoring and Acceptance Theory, the mindfulness facet *attentional monitoring* enhances cognitive processing but can also increase emotional reactivity in the absence of the second mindfulness facet, *acceptance*, which tempers affective reactivity and promotes emotional stability (Lindsay and Creswell 2017). This suggests that the distinction of effects of mindfulness facets may potentially explain mixed effects found in this review. For example, some interventions may primarily enhance attentional monitoring without simultaneously cultivating acceptance, leading participants to become more reactive to their emotions and therefore more distressed. This may highlight the importance of balancing both monitoring and acceptance when tailoring MBIs for grief. However, no reviewed studies examined whether changes in specific mindfulness facets were associated with changes in mental health problems.

Finally, a potential explanation for these findings is the occurrence of meditation‐related adverse effects that could occur within MBIs. A review showed that 37% of MBI participants reported a negative impact event, often lasting less than 1 h, yet for 6%–14% of such events, it lasted longer than 1 week and was associated with hyperarousal or dissociation. As such, MBIs are similarly associated with transient distress and negative emotional impact as other psychological treatments (Britton et al. 2021). These findings challenge the assumption that MBIs are uniformly benign and suggest that, in vulnerable individuals, mindfulness practices may yield unintended or even counterproductive effects (Schellekens et al. 2021). In response to such concerns, *trauma‐sensitive mindfulness* has been developed to enhance safety and decrease risks (Treleaven 2018), which may be particularly relevant when tailoring MBIs for grief.

Ultimately, this review highlights both the promise and complexity of mindfulness as an approach to bereavement care. To clarify the potential usefulness of mindfulness in clinical practice, future research should prioritize rigorous multi‐wave longitudinal surveys and randomized controlled trials of grief‐adapted MBIs, particularly among bereaved adults with PGD. Such studies may help clarify the effects of mindfulness (facets) in bereaved adults. Moreover, we recommend explorations of the mechanisms of action of MBIs, moderators of treatment response and potential adverse outcomes in this population. Such work will be crucial in informing the development of targeted, personalized and safe interventions for bereaved individuals.

Although further research is needed and current evidence remains mixed, the present findings have some practical implications. Both self‐reported mindfulness and MBIs appear to offer some psychological benefits for those coping with bereavement. Given that MBIs emphasize nonjudgmental awareness and acceptance rather than employing cognitively demanding strategies such as restructuring dysfunctional beliefs, they may require fewer cognitive resources (Sheikhzadeh et al. 2021). MBIs could therefore serve as a useful alternative or complementary option when first‐choice treatment, CBT, is unavailable or not preferred (Bryant et al. 2024), particularly for secondary mental health problems. However, considering the inconsistent effects on prolonged grief symptoms, MBIs are currently not recommended to treat prolonged grief before further evidence of their beneficial effects has accumulated.

### 3.1 Strengths, Limitations and Directions for Future Research

This systematic review provided the first comprehensive systematic synthesis of the relationships of mindfulness with prolonged grief and secondary bereavement‐related mental health problems. However, some limitations regarding the included studies should be acknowledged. First, the review included only one randomized controlled trial, limiting the strength of current evidence and making it difficult to establish causal links between mindfulness and bereavement‐related mental health outcomes. We recommend conducting more studies with rigorous longitudinal and experimental designs. Second, participants in the included studies were predominantly female and often had experienced specific losses, such as spousal or child loss, potentially threatening the generalizability of these findings to the broader bereaved population. Future studies using more diverse and representative samples of bereaved individuals are recommended. Third, although few studies explored potential mechanisms linking self‐reported mindfulness or MBIs to bereavement‐related mental health outcomes (Emrich et al. 2023; Huang 2022; Knowles et al. 2021), research in this area remains scarce. This gap hinders a comprehensive understanding of how mindfulness exerts its effects in bereaved adults.

There were also some limitations pertaining to the review. First, this review provided a comprehensive overview of the relationship of mindfulness with prolonged grief symptoms and other bereavement‐related mental health outcomes and, as such, included diverse study designs. Due to substantial methodological variability across studies and a relatively low number of studies using similar designs, conducting a meta‐analysis was not feasible. This limited our capacity to quantify the overall magnitude of effects of self‐reported mindfulness and MBIs on mental health outcomes. Second, we only included peer‐reviewed studies published in English, potentially excluding relevant studies published in other languages and grey literature, such as dissertations, which might introduce publication or language bias.

## 4 Conclusion

Self‐reported mindfulness and MBIs may be helpful to bereaved adults by attenuating secondary bereavement‐related mental health problems such as depression and stress. However, empirical findings regarding their impact on prolonged grief symptoms are inconclusive. While MBIs show clinical potential for enhancing mental health following loss, they should not be regarded as a one‐size‐fits‐all solution. Future research must clarify the treatment mechanisms, active components and moderators that determine when and for whom self‐reported mindfulness and MBIs can effectively promote psychological adjustment among the bereaved, which may guide the development of more effective intervention strategies for distressed bereaved adults.

## Funding

This work is supported by the China Scholarship Council (CSC) under Grant No. 202406870009.

## Conflicts of Interest

The authors declare no conflicts of interest.

## Supporting information


**Table S1:** Overview of mindfulness intervention.

## Data Availability

All data are presented in the manuscript.
