# Supplementary material for: Mindfulness in Adaptation to Bereavement: A Systematic Review
Source: Clin Psychol Psychother. 2025 Dec 11;32(6):e70199. doi: 10.1002/cpp.70198 (PMC12698905; doi:10.1002/cpp.70198)
Supplement: Supplementary file 1 — Table S1: Overview of mindfulness intervention. [file CPP-32-e70199-s001.docx]

| **Table 3**  *Overview of mindfulness intervention* | | | | |
| --- | --- | --- | --- | --- |
| Author (year) | Intervention (name, duration and format) | Grief-specific adaptation | Mindfulness measure | Mindfulness changes |
| Huang (2019) | MBCT: 8-week program with weekly 2.5 hours group sessions and daily home practice (30-40 minutes per day) | Adaptation to target bereaved individuals by adding a 2-hour introductory session focused on grief acknowledgment and psycho-physical reactions to loss. | FFMQ (Baer et al., 2008) | Significant increase in mindfulness was observed following MBCT intervention (*d* = 0.80). |
| Thieleman (2014) | ATTEND (Mindfulness-Based Bereavement Care Model):  Average of 14.64 hours individual counseling per client and no fixed number of sessions | Developed specifically for traumatic grief; includes grief-focused elements such as attunement, therapeutic touch, and death education. | Not reported |  |
| Scocco (2019) | Panta Rhei: Two days mindfulness retreat | Adapted for suicide-bereaved individuals. Included grief- and guilt/shame-focused meditations (e.g., grief meditation, forgiveness, loving-kindness) and a novel “Mindful-Walking-in-Pairs” practice. | FFMQ (Baer et al., 2008);  Mindfulness subscale of SCS (Neff, 2003) | The describe facet significantly increased. No significant changes were observed in other mindfulness facets or the mindfulness subscale of the SCS. |
| Knowles (2021) | Mindfulness Training Intervention: 6-week program. Each weekly session included 10–25 minutes of in-session mindfulness practice, group discussions, and home assignments. Home practice progressed from 5 to 19 minutes of daily guided meditation. | Minimal adaptation from standard MBI; a grief-specific rationale was provided during the first session, but core mindfulness content was not modified. | EQ-D (Fresco et al., 2007) | Mindfulness training group did not significantly enhance decentering compared to progressive muscle relaxation and wait list group. |
| O'Connor (2014) | MBCT: 8 weeks (2-hour sessions) + 2 booster sessions at 3- and 6-months post-intervention | No grief-specific adaptation; minor adjustments were made for older adults (e.g., shorter sessions, focus on general negative affect), but the MBCT protocol followed the standard manual. | Not reported |  |
| Scocco (2022) | Panta Rhei: Two days (16 hours) mindfulness retreat | Adapted for suicide-bereaved individuals. Included grief- and guilt/shame-focused meditations (e.g., grief meditation, forgiveness, loving-kindness) and a novel “Mindful-Walking-in-Pairs” practice. | FFMQ (Baer et al., 2008);  Mindfulness subscale of SCS (Neff, 2003) | Mindfulness intervention group showed greater increase in observe, describe, non-judging, and non-reactivity facets but not act with awareness facet and mindfulness subscale of SCS. |
| Thieleman (2020) | Selah: Fully Inhabited Grief: 4-day residential contemplative retreat | Based on the Selah Model (Cacciatore, 2012), addressing grief in three phases: *being with grief*, *surrendering to grief*, and *doing with grief*. Activities were tailored to parental bereavement and emphasized attunement, nonjudgment, and transformation of grief. | FFMQ (Baer et al., 2008) | Intervention group showed greater improvements in describing (*η*²ₚ = .05) and act with awareness (*η*²ₚ = .08) at the post intervention and at the 5 to 9.5 weeks follow-up compared to the online support forum comparison group. |
| Bryant (2024) | MBCT: 11-session program. Each session included ≥40 minutes of mindfulness practice. Daily home practice (40 min) with guided audio. | Minimal adaptation from standard MBCT to target prolonged grief disorder (i.e., psycho-education on prolonged grief disorder, grief responses, and positive memories). | Not reported |  |
| Note: MBCT: Mindfulness-Based Cognitive Therapy; FFMQ: Five Facet Mindfulness Questionnaire; ATTEND: Attunement, Trust, Therapeutic touch, Egalitarianism, Nuance, and Death education; SCS: Self-Compassion Scale; EQ-D: Experiences Questionnaire-Decentering subscale | | | | |
